# Frailty and nutritional status as prognostic factors in older adults with heart failure: a public health perspective

**DOI:** 10.3389/fpubh.2026.1893715

**Published:** 2026-08-14

**Authors:** Jing Li, Meiling Liu, Jingyan Wang

**Affiliations:** Yuncheng Central Hospital, Shanxi Medical University, Yuncheng, Shanxi, China

**Keywords:** Cox regression, frailty, heart failure in older adults, nutritional status, prognostic factors

## Abstract

**Background:**

Heart failure is a common cardiovascular disease among older adults. Combined with frailty, it may aggravate the patient's condition and affect the prognosis. Nutritional status is important in older patients, but its relationship to frailty associated with heart failure is not fully understood.

**Methods:**

This single-center retrospective study initially screened 236 older patients with heart failure who were admitted to Yuncheng Central Hospital in Yuncheng, Shanxi Province, China, between March 2020 and February 2023. After 36 patients were excluded according to the predefined eligibility criteria, 200 patients were included in the final analysis. A two-stage grouping strategy was used. First, all included patients underwent frailty assessment: patients without frailty were assigned to the control group, whereas those with frailty were assigned to the experimental group. Prognostic outcomes were compared between these two primary groups. Second, only patients in the experimental group underwent nutritional assessment and were further divided into the normal nutrition, mild malnutrition, and moderate-to-severe malnutrition subgroups. Appropriate parametric or nonparametric tests were used for comparisons between the frailty and non-frailty groups and among the three nutritional-status subgroups. A multivariable Cox proportional hazards regression model was used to evaluate factors associated with adverse prognosis.

**Results:**

A total of 200 older patients with heart failure were included. Among them, 92 patients with frailty were assigned to the experimental group, whereas 108 patients without frailty were assigned to the control group. During the 6-month follow-up, mortality was higher in the experimental group than in the control group [4/92 (4.35%) vs. 1/108 (0.93%)], as was the readmission rate [13/92 (14.13%) vs. 2/108 (1.85%)] (both *P* < 0.05). Within the experimental group, 19 patients (20.65%) were classified into the normal nutrition subgroup, 31 (33.70%) into the mild malnutrition subgroup, and 42 (45.65%) into the moderate-to-severe malnutrition subgroup. Adverse outcomes occurred in 1/19 patients (5.26%), 5/31 patients (16.13%), and 11/42 patients (26.19%), respectively, with the incidence increasing as nutritional status worsened (*P* < 0.05). Serum albumin (ALB) and prealbumin (PAB) levels, Geriatric Nutritional Risk Index (GNRI) scores, and the proportion of patients with high-density lipoprotein (HDL) ≥ 0.8 mmol/L were lower in the two malnutrition subgroups than in the normal nutrition subgroup. These indicators were also lower in the moderate-to-severe malnutrition subgroup than in the mild malnutrition subgroup (all *P* < 0.05). Multivariable Cox proportional hazards regression analysis showed that GNRI and HDL were associated with adverse prognosis (both *P* < 0.05).

**Conclusion:**

Frailty in older patients with heart failure is detrimental to the prognosis, and nutritional status is closely related to the prognosis of frailty in older patients with heart failure, which may lead to prolonged hospitalization, increased rehospitalization rates, and an increased risk of death. Therefore, clinical practice should pay attention to the assessment of patients' nutritional status and take corresponding measures to intervene to improve the patient's prognosis.

## 1 Introduction

Heart failure (CHF) is a common and serious cardiovascular disease among older adults. As the aging of the population intensifies, the number of older patients with heart failure has gradually increased, placing great pressure on the healthcare system and social economy (1, 2). Frailty is very common among older adults and usually increases with age. This is because the physiological reserves of various system organs of the human body gradually decrease with age. This results in a reduced ability to resist stress and maintain body homeostasis, and even minor external stimuli may cause serious health problems such as disability, falls, fractures, and even death. Central dysfunction (heart failure) is also more common among older adults, and its pathogenesis is similar to that of frailty to some extent. Previous studies have found that frailty is one of the common complications in older patients with heart failure, and its clinical manifestations are mainly characterized by reduced physical activity, weight loss, and muscle weakness (3, 4). The occurrence of combined frailty will not only affect the patient's quality of life, but also aggravate the patient's condition, leading to poor prognosis.

Nutritional status is of particular importance in the older adult population, especially in older patients with heart failure. Epidemiological statistics have found that a considerable proportion of patients with heart failure suffer from malnutrition or are at nutritional risk (5, 6). Good nutritional status helps to maintain the normal function of the cardiovascular system, reduce the load on the heart, and improve the compensatory capacity of the heart, thereby playing a positive role in improving the prognosis of patients. However, frail older patients with heart failure are more likely to be malnourished, which may further aggravate their condition and affect prognosis.

Currently, evidence regarding the association between nutritional status and prognosis in older patients with heart failure and frailty remains limited. Therefore, this study aimed to evaluate prognosis in older patients with heart failure and frailty and to investigate the association between nutritional status and adverse outcomes. This study may provide a more comprehensive understanding of the prognostic significance of nutritional status in older patients with heart failure and frailty and inform appropriate clinical interventions.

## 2 Materials and methods

### 2.1 Research information

**Figure 1:**
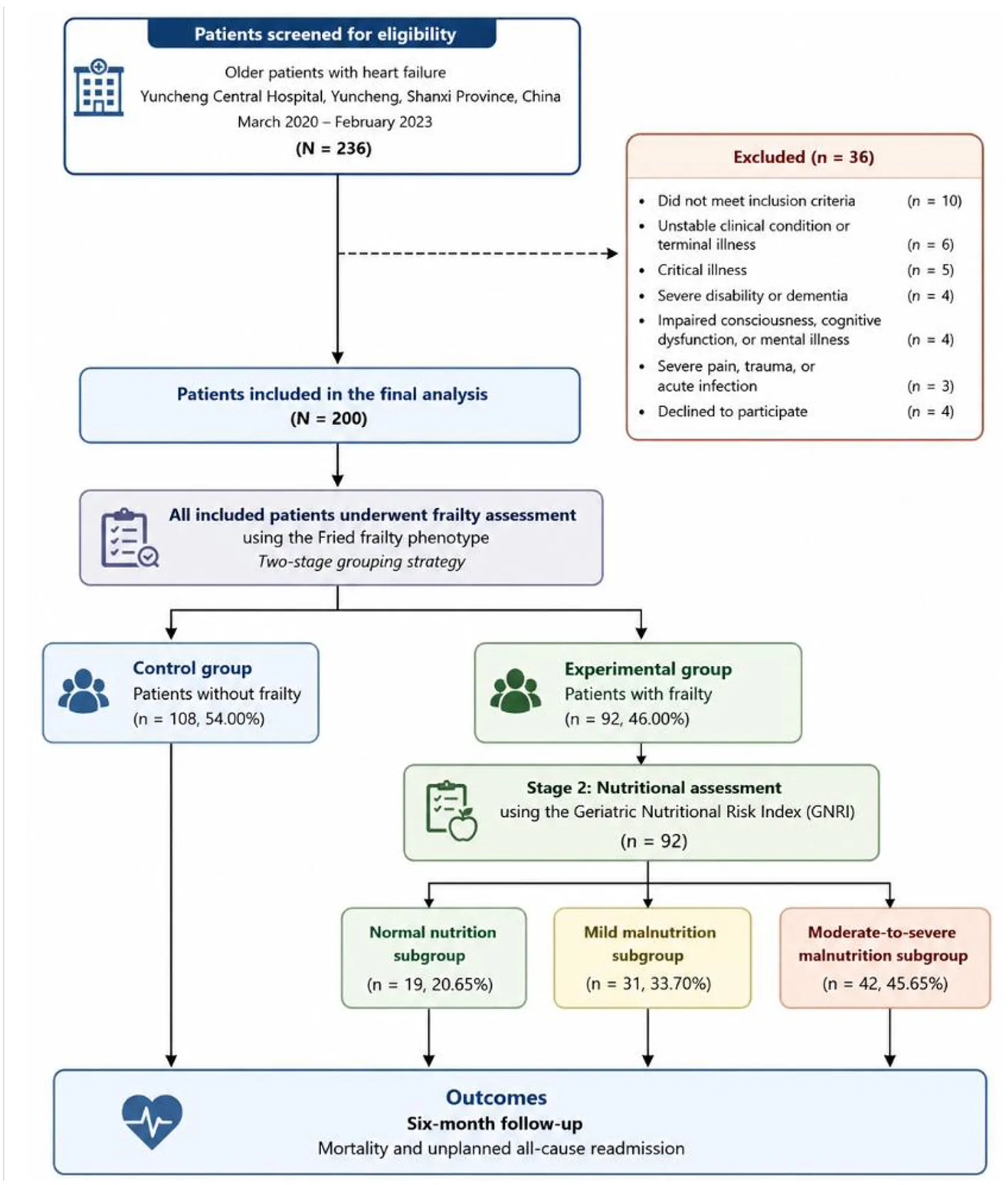
Flow diagram of patient screening, inclusion, and study grouping.

This single-center retrospective study initially screened 236 older patients with heart failure who were admitted to Yuncheng Central Hospital in Yuncheng, Shanxi Province, China, between March 2020 and February 2023. Of these, 36 patients were excluded: 10 did not meet the inclusion criteria, six had an unstable clinical condition or terminal illness, five were critically ill, four had severe disability or dementia, four had impaired consciousness, cognitive dysfunction, or mental illness, three had severe pain, trauma, or acute infection, and four declined to participate. Consequently, 200 patients were included in the final analysis. All eligible patients identified during the study period were included without additional sampling; therefore, the final cohort reasonably reflects the eligible patient population treated at this hospital during the specified period, although its representativeness for patients from other institutions or regions may be limited. All included patients underwent frailty assessment using a two-stage grouping strategy. At the first stage, 108 patients without frailty were assigned to the control group, whereas 92 patients with frailty were assigned to the experimental group. Prognostic outcomes were compared between these two primary groups. At the second stage, only the 92 patients in the experimental group underwent nutritional assessment and were further classified into the normal nutrition, mild malnutrition, and moderate-to-severe malnutrition subgroups. These three nutritional-status subgroups were secondary subgroups within the experimental group rather than additional groups in the overall cohort. All participants were fully informed of the study purpose and procedures and provided written informed consent before enrollment. The study overview flowchart is shown in Figure 1.

### 2.2 Inclusion criteria

The inclusion criteria were as follows: (1) age >60 years; (2) New York Heart Association (NYHA) functional class II–IV (7); (3) clear consciousness and the ability to communicate; (4) a clinical diagnosis of heart failure; and (5) complete clinical data. There was no restriction on sex or body mass index, and patients with overweight or obesity were not excluded solely on the basis of body weight.

The exclusion criteria were as follows: (1) unstable clinical condition or terminal illness; (2) critical illness; (3) severe disability or dementia; (4) impaired consciousness, cognitive dysfunction, or mental illness; (5) severe pain, trauma, or acute infection; and (6) refusal to participate in the study.

### 2.3 Method

Patients received routine symptomatic treatment during hospitalization, and each patient was followed up by telephone for 6 months after discharge. The frequency of follow-up is once a month, and the duration of each telephone follow-up is 15 to 20 min. The follow-up content includes recording the follow-up date, follow-up outcomes (including survival, re-hospitalization, death, and for surviving patients, recent physical conditions and daily life behaviors will also be recorded). The patient's readmission time began on the date of confirmed illness after discharge and ended on the date of unplanned all-cause readmission.

All included patients underwent physical frailty assessment using the Fried frailty phenotype (8). The assessment comprised five criteria: unintentional weight loss, exhaustion, low physical activity, weakness indicated by reduced grip strength, and slowness indicated by reduced walking speed. Each criterion was scored as 1 when present and zero when absent. Patients meeting three or more criteria were classified as frail, whereas those meeting 0–2 criteria were classified as non-frail for the present analysis. Based on this classification, patients without frailty were assigned to the control group, whereas patients with frailty were assigned to the experimental group.

The nutritional status of patients in the experimental group was assessed using the Geriatric Nutritional Risk Index (GNRI), originally developed by Bouillanne et al. (9). The GNRI was calculated as follows: GNRI = 1.489 × serum albumin (g/L) + 41.7 × weight ratio, where the weight ratio was defined as the lesser of actual body weight/ideal body weight and one. Thus, when actual body weight was equal to or lower than ideal body weight, the actual ratio was used; when actual body weight exceeded ideal body weight, the ratio was capped at one rather than allowing a value >1. This approach prevented excess body weight from artificially increasing the GNRI score. Ideal body weight was calculated using the Lorentz equations: 0.75 × height (cm)−62.5 for men and 0.60 × height (cm)−40 for women. According to the GNRI value, nutritional status was classified as normal nutrition (GNRI ≥ 98), mild malnutrition (92 ≤ GNRI < 98), moderate malnutrition (82 ≤ GNRI < 92), or severe malnutrition (GNRI < 82). For the subgroup analyses, patients with moderate and severe malnutrition were combined into a moderate-to-severe malnutrition group.

### 2.4 Observation indicators

A 5-mL fasting peripheral venous blood sample was collected from each patient and centrifuged at 3,000 rpm for 15 min to obtain serum. Serum albumin (ALB), prealbumin (PAB), and high-density lipoprotein (HDL) levels were measured using a fully automated biochemical analyzer.

### 2.5 Statistical analysis

Statistical analyses were performed using SPSS version 26.0 (Armonk, NY, USA), and GraphPad Prism (San Diego, California, USA) version 8.0 was used to prepare the figures. The statistical analyses were conducted according to the two-stage grouping structure. Prognostic outcomes were compared between the experimental and control groups, whereas nutritional indicators and adverse outcomes were compared among the three nutritional-status subgroups within the experimental group. The distribution of continuous variables was assessed using the Shapiro–Wilk test and quantile–quantile (Q–Q) plots, and homogeneity of variance was evaluated using Levene's test. Normally distributed continuous variables are presented as the mean ± standard deviation (SD). Comparisons of continuous variables between the two primary groups were performed using the independent-samples *t*-test or Welch's *t*-test, as appropriate, whereas comparisons among the three nutritional-status subgroups were performed using one-way analysis of variance. When an overall difference was statistically significant, pairwise comparisons were conducted using the Bonferroni correction. Non-normally distributed continuous variables, when applicable, are presented as the median and interquartile range and were analyzed using the Mann–Whitney *U*-test for comparisons between two groups or the Kruskal–Wallis test for comparisons among three groups. Categorical variables are presented as *n* (%) and were compared using the chi-square test or Fisher's exact test, as appropriate. Multivariable Cox proportional hazards regression analysis was used to evaluate factors associated with adverse prognosis. Given the limited number of adverse outcome events, the Cox regression model was restricted to GNRI and HDL to reduce the risk of model overfitting. All statistical tests were two-sided, and *P* < 0.05 was considered statistically significant.

## 3 Results

### 3.1 General information

During the study period, 236 older patients with heart failure were initially screened. After 36 patients were excluded according to the predefined eligibility criteria, 200 patients were included in the final analysis. Of the included patients, 92 (46.00%) were assigned to the experimental group and 108 (54.00%) to the control group. The mean age was 75.03 ± 8.44 years, and the mean body mass index was 20.94 ± 2.17 kg/m^2^. Body mass index was analyzed as a continuous variable, and no body mass index category was used as an eligibility restriction. The study population included 117 men (58.50%) and 83 women (41.50%). Regarding educational level, 88 patients (44.00%) had primary or junior high school education, whereas 112 patients (56.00%) had high school education or above. The mean disease duration was 13.45 ± 5.21 years.

### 3.2 Prognosis

**Figure 2:**
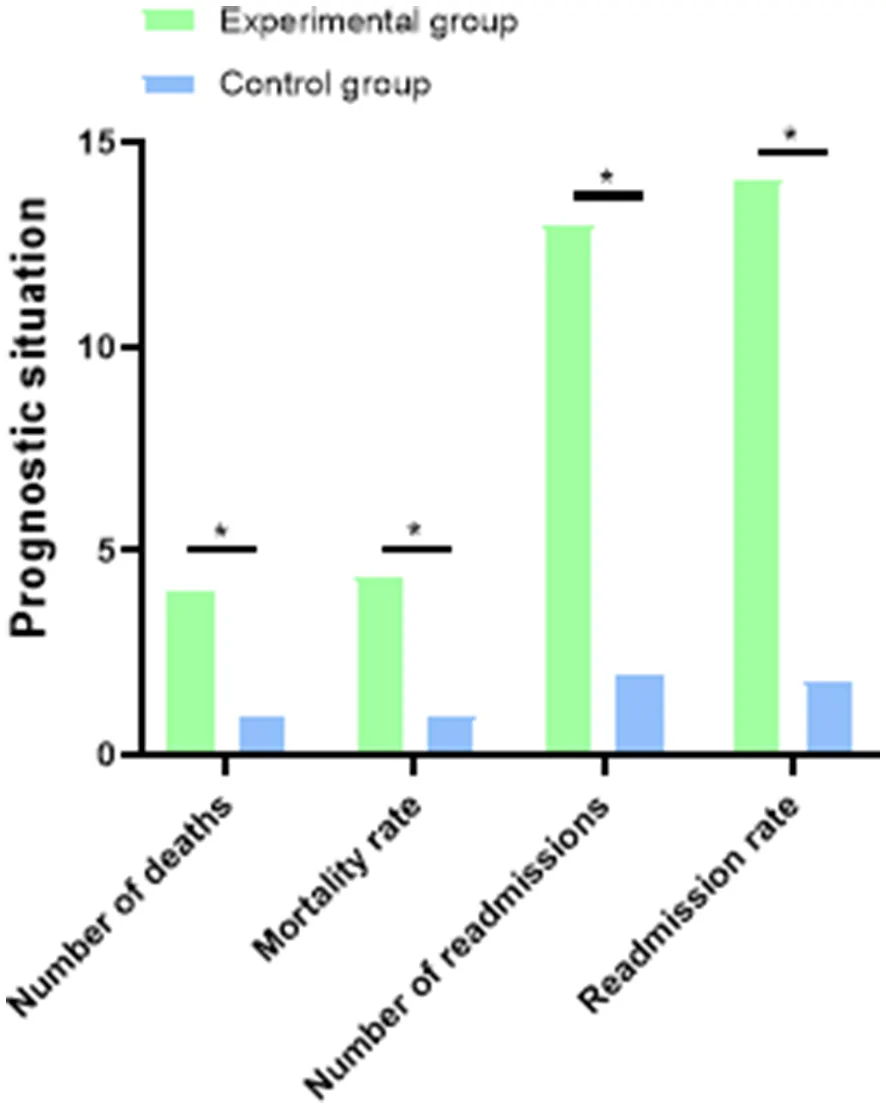
Comparison of mortality and readmission outcomes between the two groups. *P < 0.05 vs. the control group.

During the 6-month follow-up, four of 92 patients (4.35%) in the experimental group and one of 108 patients (0.93%) in the control group died. Readmission occurred in 13 of 92 patients (14.13%) in the experimental group and two of 108 patients (1.85%) in the control group. Both mortality and readmission rates were significantly higher in the experimental group than in the control group (*P* < 0.05; Figure 2).

### 3.3 Nutritional status

Within the experimental group, the 92 patients were further divided into three nutritional-status subgroups: 19 patients (20.65%) in the normal nutrition group, 31 (33.70%) in the mild malnutrition group, and 42 (45.65%) in the moderate-to-severe malnutrition group. These were secondary subgroups used only for the nutritional-status analyses. Adverse outcomes occurred in one of 19 patients (5.26%) in the normal nutrition group, five of 31 patients (16.13%) in the mild malnutrition group, and 11 of 42 patients (26.19%) in the moderate-to-severe malnutrition group. The incidence of adverse outcomes increased with worsening nutritional status (*P* < 0.05; Table 1).

**Table 1:** Comparison of adverse outcomes among the three nutritional-status subgroups within the experimental group.

Nutritional status subgroup	Normal nutrition group	Mild malnutrition group	Moderate-to-severe malnutrition group
Patients	19 (20.65)	31 (33.70)	42 (45.65)
Poor prognosis	1 (5.26)	5 (16.13)^*^	11 (26.19)^*#^

Data are presented as *n* (%). The overall comparison was performed using the chi-square test, followed by Bonferroni-adjusted pairwise comparisons when appropriate. ^*^P < 0.05 vs. the normal nutrition group; ^#^*P* < 0.05 vs. the mild malnutrition group.

### 3.4 Nutritional indicators

Among the three nutritional-status subgroups within the experimental group, serum ALB and PAB levels, GNRI scores, and the proportion of patients with HDL ≥ 0.8 mmol/L were lower in both malnutrition subgroups than in the normal nutrition group. These indicators were also lower in the moderate-to-severe malnutrition group than in the mild malnutrition group (*P* < 0.05; Table 2).

**Table 2:** Comparison of nutritional indicators among the three nutritional-status subgroups within the experimental group.

Nutritional indicator	Normal nutrition group	Mild malnutrition group	Moderate-to-severe malnutrition group
Patients	19 (20.65)	31 (33.70)	42 (45.65)
ALB (g/L)	39.94 ± 4.52	36.11 ± 3.52^*^	32.03 ± 2.08^*#^
PAB (g/L)	276.25 ± 12.65	248.42 ± 9.44^*^	215.03 ± 7.28^*#^
GNRI	99.16 ± 0.54	93.01 ± 2.11^*^	79.13 ± 5.73^*#^
HDL ≥ 0.8 mmol/L	15 (78.95)	24 (77.42)^*^	26 (61.90)^*#^

Continuous variables are presented as mean ± SD and were compared using one-way analysis of variance. Categorical variables are presented as *n* (%) and were compared using the chi-square test or Fisher's exact test, as appropriate. Pairwise comparisons were adjusted using the Bonferroni method. ^*^P < 0.05 vs. the normal nutrition group; ^#^*P* < 0.05 vs. the mild malnutrition group. ALB, albumin; PAB, prealbumin; GNRI, geriatric nutritional risk index; HDL, high-density lipoprotein; SD, standard deviation.

### 3.5 Multivariable Cox proportional hazards regression analysis

Multivariable Cox proportional hazards regression analysis showed that GNRI and HDL were associated with adverse prognosis (*P* < 0.05; Table 3).

**Table 3:** Multivariable Cox proportional hazards regression analysis of factors associated with adverse prognosis.

Variable	β	SE	Wald χ^2^	HR	95% CI	*P*
GNRI	0.997	0.128	11.084	2.013	1.001–4.987	0.021
HDL	1.110	0.351	16.124	2.118	1.022–4.967	0.003

β, regression coefficient; SE, standard error; HR, hazard ratio; CI, confidence interval; GNRI, Geriatric Nutritional Risk Index; HDL, high-density lipoprotein. The event modeled was adverse prognosis.

## 4 Discussion

Among people over 70 years old, the incidence rate of heart failure exceeds 10%. Despite advances in medical technology, mortality and readmission rates remain high in older patients with heart failure. This is related to factors such as multiple diseases, frailty, polypharmacy, and cognitive impairment. Patients with heart failure have a significantly increased incidence of frailty, ranging from 15 to 74%, due to limited activity and reduced muscle exercise. In addition, heart failure can also lead to insufficient blood supply to the brain, which may lead to cognitive impairment and falls, accelerating the occurrence of frailty and disability (10, 11). Frailty is a strong predictor of mortality, readmissions, and reduced quality of life in patients with chronic heart failure.

The present study used a two-stage grouping structure. At the first stage, the overall cohort was divided into the experimental and control groups according to frailty status, and prognostic outcomes were compared between these two primary groups. Recent evidence further supports the prognostic importance of frailty in this population. Kaufmann et al. (12). reported that frailty at admission was independently associated with increased mortality in older patients hospitalized with acute heart failure, whereas improvement in frailty status during hospitalization was associated with a more favorable prognosis. In addition, Lisiak et al. (13). found that nutritional risk and frailty frequently coexisted in older patients with heart failure and were associated with poorer functional status. Although significant progress has been made in the treatment of CHF in recent years, older patients with CHF often have multiple cardiovascular risk factors, coexisting geriatric syndromes, polypharmacy, and progressive functional decline, which may contribute to greater disease severity. This situation has resulted in persistently high readmission and mortality rates. Frailty is a complex clinical syndrome that refers to a state in which the body's stress response to various stimuli is reduced, resulting in reduced function. Studies have shown that the incidence of frailty increases significantly with age, and among people over 65 years old, 10 to 14% develop varying degrees of frailty (14, 15). Furthermore, frailty is considered a biological or geriatric syndrome whose main feature is increased vulnerability to internal and external stressors. This increased vulnerability increases the risk of falls, hospitalization, disability, and death. In patients with heart failure, disturbances and dysregulation of neurohormonal, metabolic, inflammatory, and immune pathways may lead to enhanced signaling of catabolic states, energy depletion, oxidative stress, and increased release of pro-inflammatory signals, thereby increasing the risk of frailty (16, 17). At the same time, epidemiological surveys show that among older adults with cardiovascular disease, the incidence of frailty can be as high as 10 to 60%. More clinical data show that patients with frailty and cardiovascular disease have a poor prognosis, although specific The numbers may vary depending on the survey respondents and instruments, but the results are still reliable and consistent with the results of this study.

Malnutrition is a comprehensive physiological state involving protein reserves, energy expenditure, and weakened immune system. The nutritional status of patients with heart failure reflects their overall health status, including physical condition, protein metabolism, and immune function (18, 19). And frailty is a reversible dynamic state. Through quality management, the debilitating phenomenon of cardiovascular disease or multiple diseases can be reversed and the poor prognosis of older patients can be improved. Polypharmacy is one of the debilitating factors in patients with heart failure. Therefore, the treatment of such patients is no longer just about adding more drugs, but taking comprehensive health management measures, such as muscle resistance exercise, increasing caloric and protein intake, and vitamin D supplementation, to improve the patient's nutritional status. Moreover, malnutrition is also one of the common complications in patients with heart failure. Fluid retention in patients with CHF may lead to gastrointestinal edema and inflammation, thereby impairing food intake and gastrointestinal nutrient absorption and contributing to malnutrition. Past studies have confirmed that malnutrition is an independent risk factor for adverse outcomes in patients with cardiovascular disease. It interacts with cardiovascular disease, forming a vicious cycle and further increasing patient mortality (20, 21). It may be that the decline in cardiac function in older patients with chronic heart failure leads to increased peripheral circulation resistance and increased pulmonary circulation pressure, which in turn causes insufficient blood perfusion in various organs, ultimately causing gastrointestinal congestion and affecting the intake of nutrients. In addition, Combined with frailty, malnutrition is more likely to occur due to factors such as aging and decreased body function. In addition, physiological mechanisms such as liver dysfunction, cytokine-induced hypercatabolism, and insulin resistance also aggravate the patient's malnutrition problem, which in turn leads to changes in systemic basal metabolism and an increase in the body's energy consumption. This situation of high energy consumption and low nutritional intake makes the patient's malnutrition problem increasingly serious. The worsening of malnutrition often leads to increased fluid retention in older patients with chronic heart failure, and also affects the molecular synthesis process, causing patients to fall into an irreversible vicious cycle (22, 23). At the second stage, the 92 patients in the experimental group were further divided into three nutritional-status subgroups. Within the experimental group, worsening nutritional status was associated with a higher incidence of adverse outcomes, and patients in the moderate-to-severe malnutrition subgroup had the highest incidence of poor outcomes. These findings indicate that nutritional status is closely associated with prognosis among older patients with heart failure and frailty and support the need for timely nutritional assessment and intervention in clinical practice. Consistent with the present findings, Chen et al. (24). reported in a nationwide prospective cohort that lower GNRI was associated with increased risks of mortality, major adverse cardiovascular events, and heart failure rehospitalization, and provided additional prognostic value beyond body mass index or serum albumin alone. Moreover, serum ALB, PAB, and HDL levels were compared among the three nutritional-status subgroups within the experimental group, and the observed differences were generally consistent with previous findings. Serum ALB is synthesized by liver parenchymal cells and is one of the important nutrients in the human body. PAB is a liver glycoprotein that reflects nutritional protein indicators in the body. Research by Zhang Jun et al. found that serum ALB levels in heart failure patients with good prognosis were significantly higher than those in patients with poor prognosis, and higher serum ALB levels were associated with more favorable long-term outcomes in older patients with heart failure. Heart failure and frailty are linked by similar mechanisms, including activation of oxidative stress and an increase in pro-inflammatory factors, and nutritional deficiencies may exacerbate this process. Improving nutritional status can combat oxidative stress and inflammation. For example, some studies have shown that vitamin E can reduce the degree of oxidative stress response, while vitamin D supplementation may increase the level of the anti-inflammatory cytokine interleukin-10 (IL-10) in patients with heart failure (25, 26). In other words, the treatment of older patients with heart failure and frailty should not be limited to traditional management, but should focus on ensuring appropriate caloric intake and a comprehensive and balanced diet to improve the occurrence of adverse prognosis. However, the specific types and types of nutritional supplements. The amount needs more clinical research to further explore and confirm.

Compared with previous studies, the present findings further highlight the adverse prognostic significance of coexisting frailty in older patients with heart failure and demonstrate that nutritional status is also associated with clinical outcomes, thereby providing additional evidence to support comprehensive clinical management. Nevertheless, several limitations should be acknowledged. First, the relatively small sample size and limited number of adverse outcome events may have reduced the stability and generalizability of the findings. Although age and sex may influence both nutritional status and prognosis, these variables were not included in the Cox regression model because adding further covariates in the context of a limited number of events could have increased the risk of overfitting and unstable estimates. Therefore, residual confounding by age, sex, and other clinical factors cannot be excluded. For the same reason, additional *post hoc* subgroup analyses according to age, sex, heart failure duration, or body mass index category were not performed. These analyses were not prespecified, and further division of the sample would have resulted in small subgroup sizes and few outcome events, increasing the risks of unstable estimates, multiple-testing error, and chance findings. Second, this was a single-center retrospective study conducted at Yuncheng Central Hospital. Although all eligible patients identified during the study period were included without additional sampling, the cohort primarily reflects patients treated at this institution and may not be fully representative of older patients with heart failure in other institutions, regions, or healthcare settings. Furthermore, the retrospective design may have resulted in incomplete data collection and potential selection bias. Future prospective multicenter studies with larger sample sizes and more outcome events are therefore needed to validate these findings using models adjusted for age, sex, and other clinically relevant covariates. Overall, this study provides preliminary evidence supporting an association between nutritional status and prognosis in older patients with heart failure and frailty.

In summary, frailty was associated with poorer prognosis in older patients with heart failure, and nutritional status was also associated with adverse outcomes in patients with frailty, including higher risks of readmission and death. Therefore, clinical practice should pay attention to the assessment of patients' nutritional status and take corresponding measures to intervene to improve the patient's prognosis.

## Data Availability

The original contributions presented in the study are included in the article/supplementary material, further inquiries can be directed to the corresponding author.
